# Editorial: Novel strategies targeting obesity and metabolic diseases, volume II

**DOI:** 10.3389/fphys.2024.1342943

**Published:** 2024-01-30

**Authors:** Xinran Ma, Dechun Feng, Yan Lu, Nuo Sun, Jiqiu Wang, Lingyan Xu

**Affiliations:** ^1^ Shanghai Key Laboratory of Regulatory Biology, Institute of Biomedical Sciences and School of Life Sciences, East China Normal University, Shanghai, China; ^2^ National Institute on Alcohol Abuse and Alcoholism (NIAAA), National Institutes of Health (NIH), Bethesda, MD, United States; ^3^ Institute of Metabolism and Regenerative Medicine, Shanghai Sixth People’s Hospital Affiliated to Shanghai Jiao Tong University School of Medicine, Shanghai, China; ^4^ Wexner Medical Center, The Ohio State University, Columbus, OH, United States; ^5^ National Key Laboratory for Medical Genomes, Department of Endocrinology and Metabolism, China National Research Center for Metabolic Diseases, Ruijin Hospital, Shanghai Jiao Tong University School of Medicine (SJTUSM), Shanghai, China

**Keywords:** obesity, metabolic diseases, adipose tissue, liver, muscle

Obesity and metabolic diseases including type 2 diabetes, cardiovascular disease, atherosclerosis, non-alcoholic fatty liver diseases (NAFLD), and obstructive sleep apnea threaten human health and life quality. In the second volume of this Frontiers Research Topic, we pay special attention to recent clinic advances and focusing on the contribution of previously unappreciated cell types, including macrophages and vascular endothelial cells, to these metabolic diseases.

Firstly, maintaining systemic homeostasis needs the coordination of different organs in the body. Adipose tissue and liver produce and secrete specific organokines such as adipokines and hepatokines in response to nutritional and environmental stimuli, while dysregulation of these organokines causes metabolic diseases, thus making them potential clinical biomarkers and therapeutic targets. As adipokines, Nrg4 increases brown adipose tissue activity, drive the browning of white adipose tissue, prevent lipogenesis in the liver, and improve insulin sensitivity, while adipsin increases adipogenesis and lipid accumulation. Guo et al. indicated that adiposity measurements, including waist circumference, visceral fat level, and muscle mass to visceral fat ratio are closely linked with circulating Nrg4 and adiposin levels in obese adults in a cohort of 1,212 subjects, thus provides clinical relevance of these adipokines with metabolic diseases. Meanwhile, Adropin is a hepatokine that improves glucose homeostasis, dyslipidemia, obesity-associated hyperinsulinemia, and energy homeostasis. Li et al. demonstrated that metabolic dysfunction-associated fatty liver disease (MAFLD) is correlated with adropin levels. Adropin plasma levels in MALFD and type 2 diabetes patients were lower than healthy control subjects. Serum adropin concentrations were negatively correlated with intrahepatic triglyceride, total cholesterol, and NAFLD activity score.

Secondly, external interventions like physical activity, provide valuable therapeutic opportunities to control body weights and reduce the risk of cardiovascular diseases, while different exercise protocols may lead to various outcomes. Amaro-Gahete et al. performed a pilot study with 12-week concurrent training intervention in 12 obese men and found significant decrease in weight, body mass index, fat mass, blood pressure and cardiometabolic risk. Meanwhile, Wang et al. performed meta-analysis on eleven randomized controlled trials (RCT) with 393 subjects comparing Pilates with other physical exercises or without any intervention. The results showed that Pilates dramatically reduces body weight, BMI, and body fat percentage in adults with overweight or obesity. In addition, Nakamura et al. developed myogenetic oligodeoxynucleotides (myoDNs), which are 18-base single-strand DNAs that promote myoblast differentiation by targeting nucleolin. They applied a myoDN, iSN04, to myoblasts isolated from healthy subjects and patients with type 1 or type 2 diabetes. iSN04 treatment improved differentiation of myoblasts from diabetic patients by downregulating myostatin and interleukin-8. These studies confirmed the importance of exercise in improving metabolic health and possibility of iSN04 as a nucleic acid drug targeting myoblasts for the prevention and treatment of muscle wasting in patients with diabetes.

In addition, bariatric surgery has been shown to effectively reduce weight and obesity-related comorbidities. Obstructive sleep apnea (OSA) is a sleep-related breathing disorder and an independent risk factor for cardiovascular diseases. Chen et al. performed a cross-sectional study involving 123 metabolically healthy obese patients and 200 age- and BMI-matched metabolically unhealthy obese patients to estimate the prevalence of OSA at baseline, as well as a retrospective study including 67 patients who underwent laparoscopic sleeve gastrectomy to evaluate the remission of OSA. The results suggested that, in patients with obesity, metabolic syndrome does not add extra risk for the prevalence or severity of OSA, while both metabolically healthy and unhealthy obese patients could benefit equally from laparoscopic sleeve gastrectomy in terms of weight loss and obstructive sleep apnea remission, suggesting bariatric surgery is a promising surgery for obesity and metabolic diseases.

Thirdly, metabolic organs consist of numerous cell types that play vital roles in the pathogenesis of metabolic diseases. Macrophage is the predominant immune cell type in metabolic tissues and arteries and plays vital roles in the progression of obesity, liver diseases and atherosclerosis. Cui et al. demonstrated that the orphan G protein-coupled receptor G2A modulates lipid metabolism and atherosclerosis in low-density lipoprotein receptor deficient (Ldlr^−/−^) rats as shown by exacerbated atherosclerotic plaques in G2a^−/−^Ldlr^−/−^ double knockout rats, together with increased oxidative stress and macrophage accumulation due to increased migration and reduced apoptosis via PI3K/AKT pathway. Meanwhile, Non-alcoholic steatohepatitis (NASH) is an inflammatory disorder that is characterized by chronic activation of the hepatic inflammatory response and subsequent liver damage. Wang et al. demonstrated that the Nobiletin, a natural polymethoxylated flavone and a reported retinoic-acid-related orphan receptor α (RORα) activator, reduced the infiltration of macrophages and neutrophils and promoted M1 to M2 macrophage polarization via Krüppel-like factor 4 (KLF4) in the liver in MCD fed mice. In addition, diabetes exacerbates brain damage in cerebral ischemic stroke. Guo et al. found dysfunctional neovascularization with activated Jagged1-Notch1 signaling in the cerebrovasculature before cerebral ischemia in diabetic rats compared with non-diabetic rats, as well as delayed angiogenesis and suppressed Jagged1-Notch1 signaling after ischemic stroke. The dynamic regulation of Jagged1-Notch1 signaling is vital for diabetes-related cerebral microvasculature dysfunction after ischemic stroke. These results revealed the unappreciated roles of macrophages or endothelial cells in metabolic diseases.

Last but not the least, the complexity of physiology and architecture of liver is highly related to spatial compartmentalization known as liver zonation. Cunningham et al. reviewed recent advances in examining liver zonation and elucidating the regulatory mechanisms via single cell analysis and imaging technologies. Understanding the spatial organization of metabolism is vital to extend our knowledge of liver disease and to provide targeted therapeutic avenues.

In conclusion, the current Research Topic provides comprehensive and in-depth understandings on diagnostic biomarkers, clinical advances, unappreciated cell types and molecular mechanisms, thus overall provide latest trends and technique advances towards therapeutic strategies to combat obesity and metabolic diseases.

